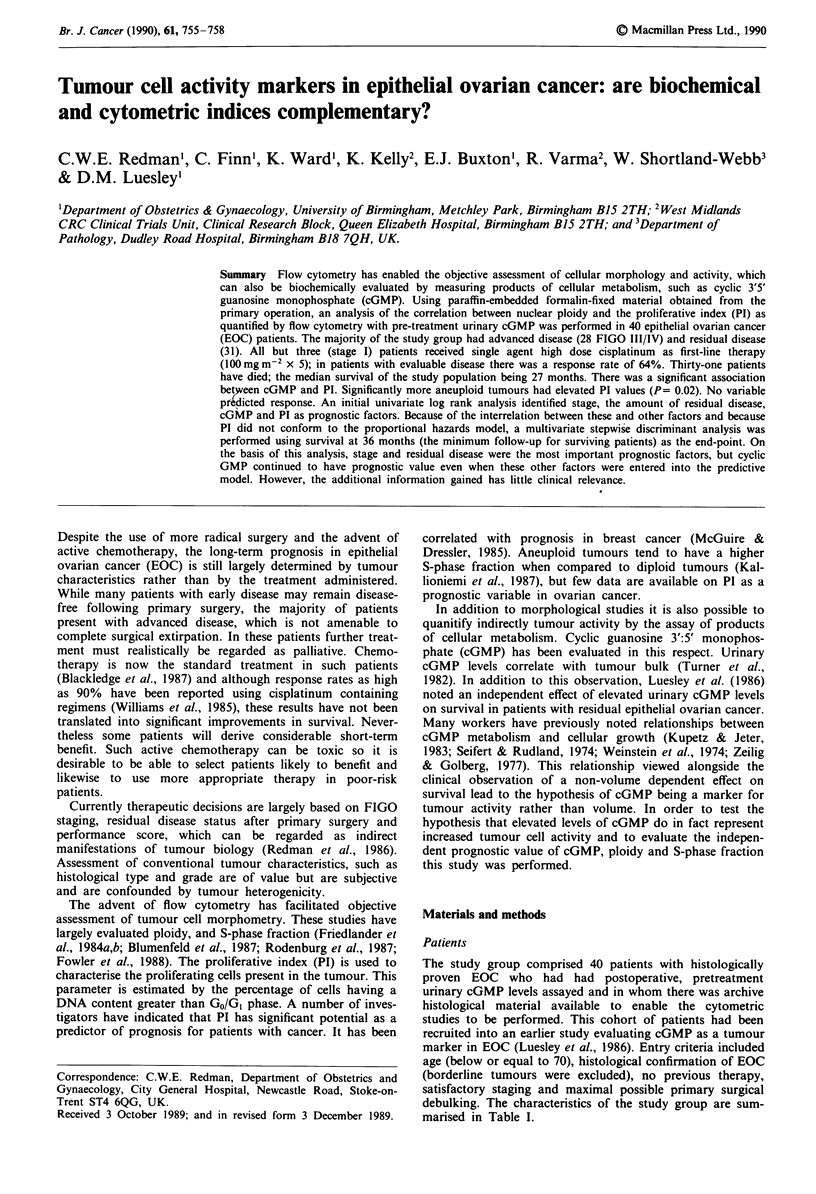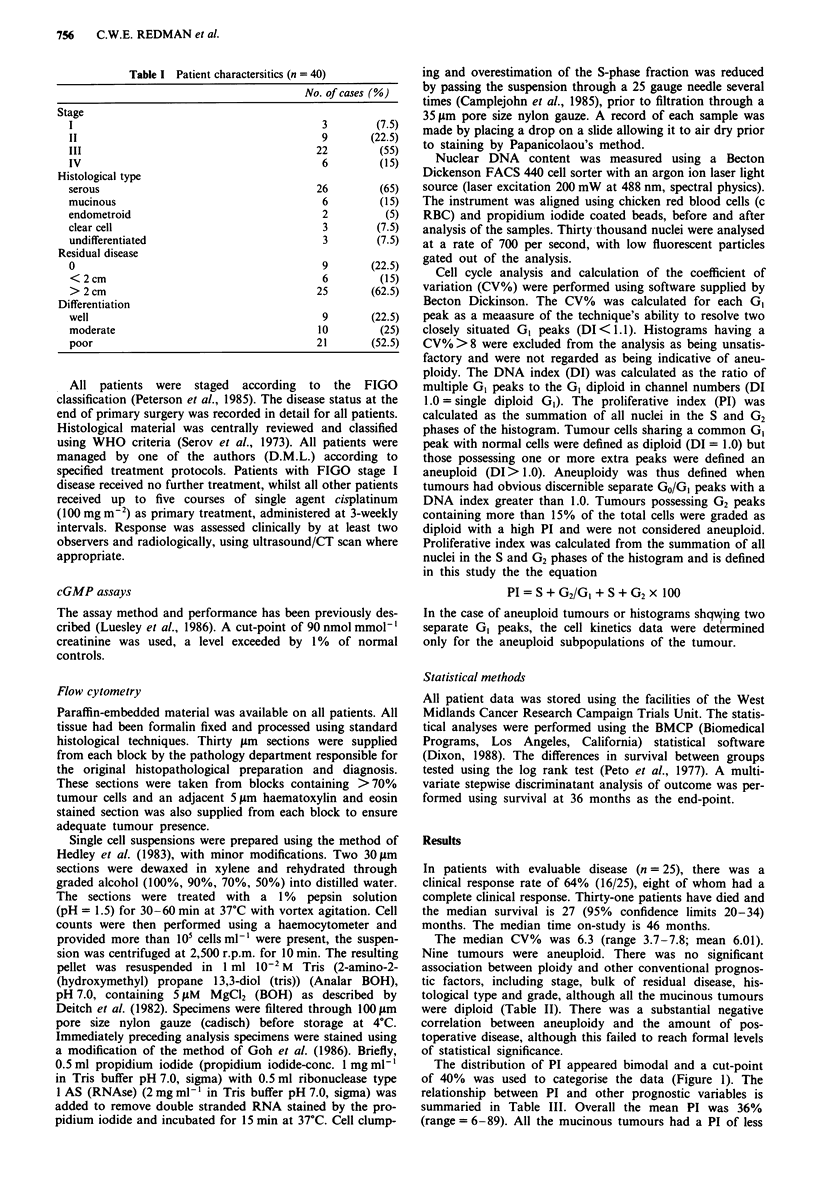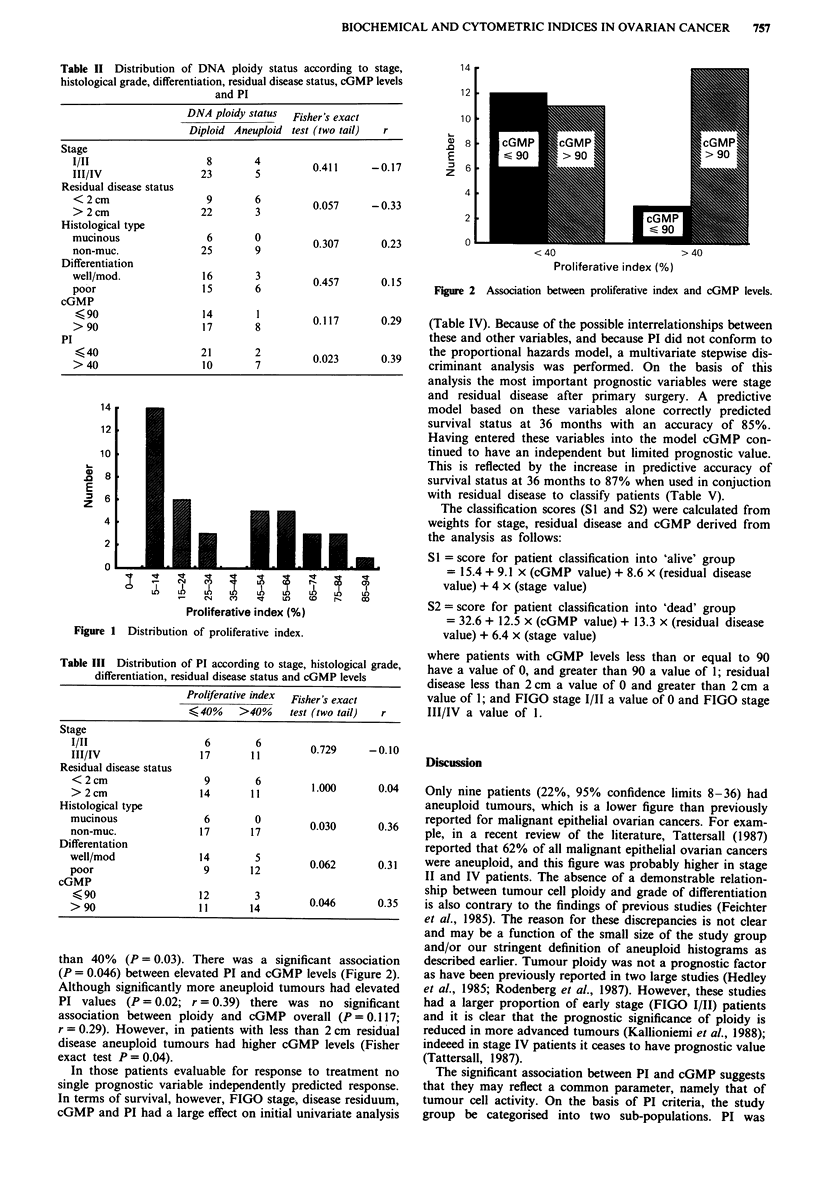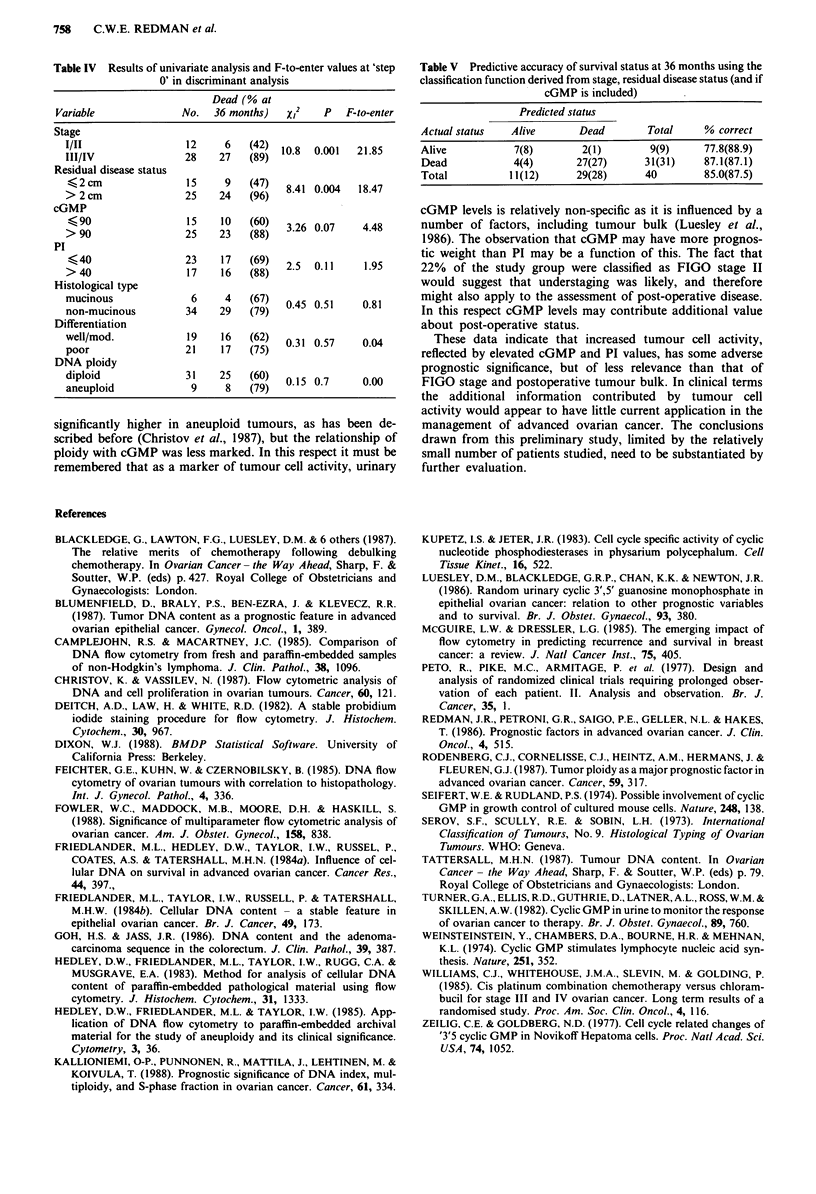# Tumour cell activity markers in epithelial ovarian cancer: are biochemical and cytometric indices complementary?

**DOI:** 10.1038/bjc.1990.168

**Published:** 1990-05

**Authors:** C. W. Redman, C. Finn, K. Ward, K. Kelly, E. J. Buxton, R. Varma, W. Shortland-Webb, D. M. Luesley

**Affiliations:** Department of Obstetrics & Gynaecology, University of Birmingham, UK.

## Abstract

Flow cytometry has enabled the objective assessment of cellular morphology and activity, which can also be biochemically evaluated by measuring products of cellular metabolism, such as cyclic 3'5' guanosine monophosphate (cGMP). Using paraffin-embedded formalin-fixed material obtained from the primary operation, an analysis of the correlation between nuclear ploidy and the proliferative index (PI) as quantified by flow cytometry with pre-treatment urinary cGMP was performed in 40 epithelial ovarian cancer (EOC) patients. The majority of the study group had advanced disease (28 FIGO III/IV) and residual disease (31). All but three (stage I) patients received single agent high dose cisplatinum as first-line therapy (100 mg m-2 x 5); in patients with evaluable disease there was a response rate of 64%. Thirty-one patients have died; the median survival of the study population being 27 months. There was a significant association between cGMP and PI. Significantly more aneuploid tumours had elevated PI values (P = 0.02). No variable predicted response. An initial univariate log rank analysis identified stage, the amount of residual disease, cGMP and PI as prognostic factors. Because of the interrelation between these and other factors and because PI did not conform to the proportional hazards model, a multivariate stepwise discriminant analysis was performed using survival at 36 months (the minimum follow-up for surviving patients) as the end-point. On the basis of this analysis, stage and residual disease were the most important prognostic factors, but cyclic GMP continued to have prognostic value even when these other factors were entered into the predictive model. However, the additional information gained has little clinical relevance.


Br. J. Cancer (1990), 61, 755–758 © Macmillan Press Ltd., 1990

### Tumour cell activity markers in epithelial ovarian cancer: are biochemical and cytometric indices complementary?

C.W.E. Redman[1], C. Finn[1], K. Ward[1], K. Kelly[2], E.J. Buxton[1], R. Varma[2], W. Shortland-Webb[3] & D.M. Luesley[1]

[1]*Department of Obstetrics & Gynaecology, University of Birmingham, Metchley Park, Birmingham B15 2TH;* [2]*West Midlands CRC Clinical Trials Unit, Clinical Research Block, Queen Elizabeth Hospital, Birmingham B15 2TH; and* [3]*Department of Pathology, Dudley Road Hospital, Birmingham B18 7QH, UK.*

**Summary** Flow cytometry has enabled the objective assessment of cellular morphology and activity, which can also be biochemically evaluated by measuring products of cellular metabolism, such as cyclic 3'5' guanosine monophosphate (cGMP). Using paraffin-embedded formalin-fixed material obtained from the primary operation, an analysis of the correlation between nuclear ploidy and the proliferative index (PI) as quantified by flow cytometry with pre-treatment urinary cGMP was performed in 40 epithelial ovarian cancer (EOC) patients. The majority of the study group had advanced disease (28 FIGO III/IV) and residual disease (31). All but three (stage I) patients received single agent high dose cisplatinum as first-line therapy ($100 \text{ mg m}^{-2} \times 5$); in patients with evaluable disease there was a response rate of 64%. Thirty-one patients have died; the median survival of the study population being 27 months. There was a significant association between cGMP and PI. Significantly more aneuploid tumours had elevated PI values ($P = 0.02$). No variable predicted response. An initial univariate log rank analysis identified stage, the amount of residual disease, cGMP and PI as prognostic factors. Because of the interrelation between these and other factors and because PI did not conform to the proportional hazards model, a multivariate stepwise discriminant analysis was performed using survival at 36 months (the minimum follow-up for surviving patients) as the end-point. On the basis of this analysis, stage and residual disease were the most important prognostic factors, but cyclic GMP continued to have prognostic value even when these other factors were entered into the predictive model. However, the additional information gained has little clinical relevance.

Despite the use of more radical surgery and the advent of active chemotherapy, the long-term prognosis in epithelial ovarian cancer (EOC) is still largely determined by tumour characteristics rather than by the treatment administered. While many patients with early disease may remain disease-free following primary surgery, the majority of patients present with advanced disease, which is not amenable to complete surgical extirpation. In these patients further treatment must realistically be regarded as palliative. Chemotherapy is now the standard treatment in such patients (Blackledge *et al.*, 1987) and although response rates as high as 90% have been reported using cisplatinum containing regimens (Williams *et al.*, 1985), these results have not been translated into significant improvements in survival. Nevertheless some patients will derive considerable short-term benefit. Such active chemotherapy can be toxic so it is desirable to be able to select patients likely to benefit and likewise to use more appropriate therapy in poor-risk patients.

Currently therapeutic decisions are largely based on FIGO staging, residual disease status after primary surgery and performance score, which can be regarded as indirect manifestations of tumour biology (Redman *et al.*, 1986). Assessment of conventional tumour characteristics, such as histological type and grade are of value but are subjective and are confounded by tumour heterogenicity.

The advent of flow cytometry has facilitated objective assessment of tumour cell morphometry. These studies have largely evaluated ploidy, and S-phase fraction (Friedlander *et al.*, 1984*a,b*; Blumenfeld *et al.*, 1987; Rodenburg *et al.*, 1987; Fowler *et al.*, 1988). The proliferative index (PI) is used to characterise the proliferating cells present in the tumour. This parameter is estimated by the percentage of cells having a DNA content greater than $G_0/G_1$ phase. A number of investigators have indicated that PI has significant potential as a predictor of prognosis for patients with cancer. It has been correlated with prognosis in breast cancer (McGuire & Dressler, 1985). Aneuploid tumours tend to have a higher S-phase fraction when compared to diploid tumours (Kallioniemi *et al.*, 1987), but few data are available on PI as a prognostic variable in ovarian cancer.

In addition to morphological studies it is also possible to quanitify indirectly tumour activity by the assay of products of cellular metabolism. Cyclic guanosine 3':5' monophosphate (cGMP) has been evaluated in this respect. Urinary cGMP levels correlate with tumour bulk (Turner *et al.*, 1982). In addition to this observation, Luesley *et al.* (1986) noted an independent effect of elevated urinary cGMP levels on survival in patients with residual epithelial ovarian cancer. Many workers have previously noted relationships between cGMP metabolism and cellular growth (Kupetz & Jeter, 1983; Seifert & Rudland, 1974; Weinstein *et al.*, 1974; Zeilig & Golberg, 1977). This relationship viewed alongside the clinical observation of a non-volume dependent effect on survival lead to the hypothesis of cGMP being a marker for tumour activity rather than volume. In order to test the hypothesis that elevated levels of cGMP do in fact represent increased tumour cell activity and to evaluate the independent prognostic value of cGMP, ploidy and S-phase fraction this study was performed.

### Materials and methods

### *Patients*

The study group comprised 40 patients with histologically proven EOC who had had postoperative, pretreatment urinary cGMP levels assayed and in whom there was archive histological material available to enable the cytometric studies to be performed. This cohort of patients had been recruited into an earlier study evaluating cGMP as a tumour marker in EOC (Luesley *et al.*, 1986). Entry criteria included age (below or equal to 70), histological confirmation of EOC (borderline tumours were excluded), no previous therapy, satisfactory staging and maximal possible primary surgical debulking. The characteristics of the study group are summarised in Table I.

Correspondence: C.W.E. Redman, Department of Obstetrics and Gynaecology, City General Hospital, Newcastle Road, Stoke-on-Trent ST4 6QG, UK.
Received 3 October 1989; and in revised form 3 December 1989.

**Table I** Patient charactersitics ($n = 40$)

| | *No. of cases* | *(%)* |
|---|---|---|
| Stage | | |
| I | 3 | (7.5) |
| II | 9 | (22.5) |
| III | 22 | (55) |
| IV | 6 | (15) |
| Histological type | | |
| serous | 26 | (65) |
| mucinous | 6 | (15) |
| endometroid | 2 | (5) |
| clear cell | 3 | (7.5) |
| undifferentiated | 3 | (7.5) |
| Residual disease | | |
| 0 | 9 | (22.5) |
| < 2 cm | 6 | (15) |
| > 2 cm | 25 | (62.5) |
| Differentiation | | |
| well | 9 | (22.5) |
| moderate | 10 | (25) |
| poor | 21 | (52.5) |

All patients were staged according to the FIGO classification (Peterson *et al.*, 1985). The disease status at the end of primary surgery was recorded in detail for all patients. Histological material was centrally reviewed and classified using WHO criteria (Serov *et al.*, 1973). All patients were managed by one of the authors (D.M.L.) according to specified treatment protocols. Patients with FIGO stage I disease received no further treatment, whilst all other patients received up to five courses of single agent *cis*platinum (100 mg $m^{-2}$) as primary treatment, administered at 3-weekly intervals. Response was assessed clinically by at least two observers and radiologically, using ultrasound/CT scan where appropriate.

*cGMP assays*

The assay method and performance has been previously described (Luesley *et al.*, 1986). A cut-point of 90 nmol $mmol^{-1}$ creatinine was used, a level exceeded by 1% of normal controls.

*Flow cytometry*

Paraffin-embedded material was available on all patients. All tissue had been formalin fixed and processed using standard histological techniques. Thirty μm sections were supplied from each block by the pathology department responsible for the original histopathological preparation and diagnosis. These sections were taken from blocks containing >70% tumour cells and an adjacent 5 μm haematoxylin and eosin stained section was also supplied from each block to ensure adequate tumour presence.

Single cell suspensions were prepared using the method of Hedley *et al.* (1983), with minor modifications. Two 30 μm sections were dewaxed in xylene and rehydrated through graded alcohol (100%, 90%, 70%, 50%) into distilled water. The sections were treated with a 1% pepsin solution (pH = 1.5) for 30–60 min at 37°C with vortex agitation. Cell counts were then performed using a haemocytometer and provided more than $10^5$ cells $ml^{-1}$ were present, the suspension was centrifuged at 2,500 r.p.m. for 10 min. The resulting pellet was resuspended in 1 ml $10^{-2}$ M Tris (2-amino-2-(hydroxymethyl) propane 13,3-diol (tris)) (Analar BOH), pH 7.0, containing 5 μM $MgCl_2$ (BOH) as described by Deitch *et al.* (1982). Specimens were filtered through 100 μm pore size nylon gauze (cadisch) before storage at 4°C. Immediately preceding analysis specimens were stained using a modification of the method of Goh *et al.* (1986). Briefly, 0.5 ml propidium iodide (propidium iodide-conc. 1 mg $ml^{-1}$ in Tris buffer pH 7.0, sigma) with 0.5 ml ribonuclease type 1 AS (RNAse) (2 mg $ml^{-1}$ in Tris buffer pH 7.0, sigma) was added to remove double stranded RNA stained by the propidium iodide and incubated for 15 min at 37°C. Cell clumping and overestimation of the S-phase fraction was reduced by passing the suspension through a 25 gauge needle several times (Camplejohn *et al.*, 1985), prior to filtration through a 35 μm pore size nylon gauze. A record of each sample was made by placing a drop on a slide allowing it to air dry prior to staining by Papanicolaou's method.

Nuclear DNA content was measured using a Becton Dickenson FACS 440 cell sorter with an argon ion laser light source (laser excitation 200 mW at 488 nm, spectral physics). The instrument was aligned using chicken red blood cells (c RBC) and propidium iodide coated beads, before and after analysis of the samples. Thirty thousand nuclei were analysed at a rate of 700 per second, with low fluorescent particles gated out of the analysis.

Cell cycle analysis and calculation of the coefficient of variation (CV%) were performed using software supplied by Becton Dickinson. The CV% was calculated for each $G_1$ peak as a meaasure of the technique's ability to resolve two closely situated $G_1$ peaks (DI < 1.1). Histograms having a CV% > 8 were excluded from the analysis as being unsatisfactory and were not regarded as being indicative of aneuploidy. The DNA index (DI) was calculated as the ratio of multiple $G_1$ peaks to the $G_1$ diploid in channel numbers (DI 1.0 = single diploid $G_1$). The proliferative index (PI) was calculated as the summation of all nuclei in the S and $G_2$ phases of the histogram. Tumour cells sharing a common $G_1$ peak with normal cells were defined as diploid (DI = 1.0) but those possessing one or more extra peaks were defined an aneuploid (DI > 1.0). Aneuploidy was thus defined when tumours had obvious discernible separate $G_0/G_1$ peaks with a DNA index greater than 1.0. Tumours possessing $G_2$ peaks containing more than 15% of the total cells were graded as diploid with a high PI and were not considered aneuploid. Proliferative index was calculated from the summation of all nuclei in the S and $G_2$ phases of the histogram and is defined in this study the the equation

$$PI = S + G_2/G_1 + S + G_2 \times 100$$

In the case of aneuploid tumours or histograms showing two separate $G_1$ peaks, the cell kinetics data were determined only for the aneuploid subpopulations of the tumour.

*Statistical methods*

All patient data was stored using the facilities of the West Midlands Cancer Research Campaign Trials Unit. The statistical analyses were performed using the BMCP (Biomedical Programs, Los Angeles, California) statistical software (Dixon, 1988). The differences in survival between groups tested using the log rank test (Peto *et al.*, 1977). A multivariate stepwise discriminatant analysis of outcome was performed using survival at 36 months as the end-point.

### Results

In patients with evaluable disease ($n = 25$), there was a clinical response rate of 64% (16/25), eight of whom had a complete clinical response. Thirty-one patients have died and the median survival is 27 (95% confidence limits 20–34) months. The median time on-study is 46 months.

The median CV% was 6.3 (range 3.7–7.8; mean 6.01). Nine tumours were aneuploid. There was no significant association between ploidy and other conventional prognostic factors, including stage, bulk of residual disease, histological type and grade, although all the mucinous tumours were diploid (Table II). There was a substantial negative correlation between aneuploidy and the amount of postoperative disease, although this failed to reach formal levels of statistical significance.

The distribution of PI appeared bimodal and a cut-point of 40% was used to categorise the data (Figure 1). The relationship between PI and other prognostic variables is summaried in Table III. Overall the mean PI was 36% (range = 6–89). All the mucinous tumours had a PI of less

**Table II** Distribution of DNA ploidy status according to stage, histological grade, differentiation, residual disease status, cGMP levels and PI

| | *DNA ploidy status* | | *Fisher's exact test (two tail)* | *r* |
|---|---|---|---|---|
| | *Diploid* | *Aneuploid* | | |
| Stage | | | | |
| I/II | 8 | 4 | 0.411 | −0.17 |
| III/IV | 23 | 5 | | |
| Residual disease status | | | | |
| < 2 cm | 9 | 6 | 0.057 | −0.33 |
| > 2 cm | 22 | 3 | | |
| Histological type | | | | |
| mucinous | 6 | 0 | 0.307 | 0.23 |
| non-muc. | 25 | 9 | | |
| Differentiation | | | | |
| well/mod. | 16 | 3 | 0.457 | 0.15 |
| poor | 15 | 6 | | |
| cGMP | | | | |
| ⩽90 | 14 | 1 | 0.117 | 0.29 |
| > 90 | 17 | 8 | | |
| PI | | | | |
| ⩽40 | 21 | 2 | 0.023 | 0.39 |
| > 40 | 10 | 7 | | |

**Figure 1** Distribution of proliferative index.

**Table III** Distribution of PI according to stage, histological grade, differentiation, residual disease status and cGMP levels

| | *Proliferative index* | | *Fisher's exact test (two tail)* | *r* |
|---|---|---|---|---|
| | *⩽40%* | *>40%* | | |
| Stage | | | | |
| I/II | 6 | 6 | 0.729 | −0.10 |
| III/IV | 17 | 11 | | |
| Residual disease status | | | | |
| < 2 cm | 9 | 6 | 1.000 | 0.04 |
| > 2 cm | 14 | 11 | | |
| Histological type | | | | |
| mucinous | 6 | 0 | 0.030 | 0.36 |
| non-muc. | 17 | 17 | | |
| Differentation | | | | |
| well/mod | 14 | 5 | 0.062 | 0.31 |
| poor | 9 | 12 | | |
| cGMP | | | | |
| ⩽90 | 12 | 3 | 0.046 | 0.35 |
| > 90 | 11 | 14 | | |

than 40% ($P = 0.03$). There was a significant association ($P = 0.046$) between elevated PI and cGMP levels (Figure 2). Although significantly more aneuploid tumours had elevated PI values ($P = 0.02$; $r = 0.39$) there was no significant association between ploidy and cGMP overall ($P = 0.117$; $r = 0.29$). However, in patients with less than 2 cm residual disease aneuploid tumours had higher cGMP levels (Fisher exact test $P = 0.04$).

In those patients evaluable for response to treatment no single prognostic variable independently predicted response. In terms of survival, however, FIGO stage, disease residuum, cGMP and PI had a large effect on initial univariate analysis

**Figure 2** Association between proliferative index and cGMP levels.

(Table IV). Because of the possible interrelationships between these and other variables, and because PI did not conform to the proportional hazards model, a multivariate stepwise discriminant analysis was performed. On the basis of this analysis the most important prognostic variables were stage and residual disease after primary surgery. A predictive model based on these variables alone correctly predicted survival status at 36 months with an accuracy of 85%. Having entered these variables into the model cGMP continued to have an independent but limited prognostic value. This is reflected by the increase in predictive accuracy of survival status at 36 months to 87% when used in conjuction with residual disease to classify patients (Table V).

The classification scores (S1 and S2) were calculated from weights for stage, residual disease and cGMP derived from the analysis as follows:

S1 = score for patient classification into 'alive' group

= 15.4 + 9.1 × (cGMP value) + 8.6 × (residual disease value) + 4 × (stage value)

S2 = score for patient classification into 'dead' group

= 32.6 + 12.5 × (cGMP value) + 13.3 × (residual disease value) + 6.4 × (stage value)

where patients with cGMP levels less than or equal to 90 have a value of 0, and greater than 90 a value of 1; residual disease less than 2 cm a value of 0 and greater than 2 cm a value of 1; and FIGO stage I/II a value of 0 and FIGO stage III/IV a value of 1.

### Discussion

Only nine patients (22%, 95% confidence limits 8–36) had aneuploid tumours, which is a lower figure than previously reported for malignant epithelial ovarian cancers. For example, in a recent review of the literature, Tattersall (1987) reported that 62% of all malignant epithelial ovarian cancers were aneuploid, and this figure was probably higher in stage II and IV patients. The absence of a demonstrable relationship between tumour cell ploidy and grade of differentiation is also contrary to the findings of previous studies (Feichter *et al.*, 1985). The reason for these discrepancies is not clear and may be a function of the small size of the study group and/or our stringent definition of aneuploid histograms as described earlier. Tumour ploidy was not a prognostic factor as have been previously reported in two large studies (Hedley *et al.*, 1985; Rodenberg *et al.*, 1987). However, these studies had a larger proportion of early stage (FIGO I/II) patients and it is clear that the prognostic significance of ploidy is reduced in more advanced tumours (Kallioniemi *et al.*, 1988); indeeed in stage IV patients it ceases to have prognostic value (Tattersall, 1987).

The significant association between PI and cGMP suggests that they may reflect a common parameter, namely that of tumour cell activity. On the basis of PI criteria, the study group be categorised into two sub-populations. PI was

**Table IV** Results of univariate analysis and F-to-enter values at 'step 0' in discriminant analysis

| Variable | No. | Dead (% at 36 months) | | $\chi_1^2$ | P | F-to-enter |
|---|---|---|---|---|---|---|
| Stage | | | | | | |
| I/II | 12 | 6 | (42) | 10.8 | 0.001 | 21.85 |
| III/IV | 28 | 27 | (89) | | | |
| Residual disease status | | | | | | |
| ⩽2 cm | 15 | 9 | (47) | 8.41 | 0.004 | 18.47 |
| >2 cm | 25 | 24 | (96) | | | |
| cGMP | | | | | | |
| ⩽90 | 15 | 10 | (60) | 3.26 | 0.07 | 4.48 |
| >90 | 25 | 23 | (88) | | | |
| PI | | | | | | |
| ⩽40 | 23 | 17 | (69) | 2.5 | 0.11 | 1.95 |
| >40 | 17 | 16 | (88) | | | |
| Histological type | | | | | | |
| mucinous | 6 | 4 | (67) | 0.45 | 0.51 | 0.81 |
| non-mucinous | 34 | 29 | (79) | | | |
| Differentiation | | | | | | |
| well/mod. | 19 | 16 | (62) | 0.31 | 0.57 | 0.04 |
| poor | 21 | 17 | (75) | | | |
| DNA ploidy | | | | | | |
| diploid | 31 | 25 | (60) | 0.15 | 0.7 | 0.00 |
| aneuploid | 9 | 8 | (79) | | | |

significantly higher in aneuploid tumours, as has been described before (Christov et al., 1987), but the relationship of ploidy with cGMP was less marked. In this respect it must be remembered that as a marker of tumour cell activity, urinary cGMP levels is relatively non-specific as it is influenced by a number of factors, including tumour bulk (Luesley et al., 1986). The observation that cGMP may have more prognostic weight than PI may be a function of this. The fact that 22% of the study group were classified as FIGO stage II would suggest that understaging was likely, and therefore might also apply to the assessment of post-operative disease. In this respect cGMP levels may contribute additional value about post-operative status.

**Table V** Predictive accuracy of survival status at 36 months using the classification function derived from stage, residual disease status (and if cGMP is included)

| | Predicted status | | | |
|---|---|---|---|---|
| Actual status | Alive | Dead | Total | % correct |
| Alive | 7(8) | 2(1) | 9(9) | 77.8(88.9) |
| Dead | 4(4) | 27(27) | 31(31) | 87.1(87.1) |
| Total | 11(12) | 29(28) | 40 | 85.0(87.5) |

These data indicate that increased tumour cell activity, reflected by elevated cGMP and PI values, has some adverse prognostic significance, but of less relevance than that of FIGO stage and postoperative tumour bulk. In clinical terms the additional information contributed by tumour cell activity would appear to have little current application in the management of advanced ovarian cancer. The conclusions drawn from this preliminary study, limited by the relatively small number of patients studied, need to be substantiated by further evaluation.

### References

BLACKLEDGE, G., LAWTON, F.G., LUESLEY, D.M. & 6 others (1987). The relative merits of chemotherapy following debulking chemotherapy. In *Ovarian Cancer – the Way Ahead*, Sharp, F. & Soutter, W.P. (eds) p. 427. Royal College of Obstetricians and Gynaecologists: London.

BLUMENFIELD, D., BRALY, P.S., BEN-EZRA, J. & KLEVECZ, R.R. (1987). Tumor DNA content as a prognostic feature in advanced ovarian epithelial cancer. *Gynecol. Oncol.*, **1**, 389.

CAMPLEJOHN, R.S. & MACARTNEY, J.C. (1985). Comparison of DNA flow cytometry from fresh and paraffin-embedded samples of non-Hodgkin's lymphoma. *J. Clin. Pathol.*, **38**, 1096.

CHRISTOV, K. & VASSILEV, N. (1987). Flow cytometric analysis of DNA and cell proliferation in ovarian tumours. *Cancer*, **60**, 121.

DEITCH, A.D., LAW, H. & WHITE, R.D. (1982). A stable probidium iodide staining procedure for flow cytometry. *J. Histochem. Cytochem.*, **30**, 967.

DIXON, W.J. (1988). *BMDP Statistical Software*. University of California Press: Berkeley.

FEICHTER, G.E., KUHN, W. & CZERNOBILSKY, B. (1985). DNA flow cytometry of ovarian tumours with correlation to histopathology. *Int. J. Gynecol. Pathol.*, **4**, 336.

FOWLER, W.C., MADDOCK, M.B., MOORE, D.H. & HASKILL, S. (1988). Significance of multiparameter flow cytometric analysis of ovarian cancer. *Am. J. Obstet. Gynecol.*, **158**, 838.

FRIEDLANDER, M.L., HEDLEY, D.W., TAYLOR, I.W., RUSSEL, P., COATES, A.S. & TATERSHALL, M.H.N. (1984*a*). Influence of cellular DNA on survival in advanced ovarian cancer. *Cancer Res.*, **44**, 397.,

FRIEDLANDER, M.L., TAYLOR, I.W., RUSSELL, P. & TATERSHALL, M.H.W. (1984*b*). Cellular DNA content – a stable feature in epithelial ovarian cancer. *Br. J. Cancer*, **49**, 173.

GOH, H.S. & JASS, J.R. (1986). DNA content and the adenoma-carcinoma sequence in the colorectum. *J. Clin. Pathol.*, **39**, 387.

HEDLEY, D.W., FRIEDLANDER, M.L., TAYLOR, I.W., RUGG, C.A. & MUSGRAVE, E.A. (1983). Method for analysis of cellular DNA content of paraffin-embedded pathological material using flow cytometry. *J. Histochem. Cytochem.*, **31**, 1333.

HEDLEY, D.W., FRIEDLANDER, M.L. & TAYLOR, I.W. (1985). Application of DNA flow cytometry to paraffin-embedded archival material for the study of aneuploidy and its clinical significance. *Cytometry*, **3**, 36.

KALLIONIEMI, O-P., PUNNONEN, R., MATTILA, J., LEHTINEN, M. & KOIVULA, T. (1988). Prognostic significance of DNA index, multiploidy, and S-phase fraction in ovarian cancer. *Cancer*, **61**, 334.

KUPETZ, I.S. & JETER, J.R. (1983). Cell cycle specific activity of cyclic nucleotide phosphodiesterases in physarium polycephalum. *Cell Tissue Kinet.*, **16**, 522.

LUESLEY, D.M., BLACKLEDGE, G.R.P., CHAN, K.K. & NEWTON, J.R. (1986). Random urinary cyclic 3',5' guanosine monophosphate in epithelial ovarian cancer: relation to other prognostic variables and to survival. *Br. J. Obstet. Gynaecol.*, **93**, 380.

MCGUIRE, L.W. & DRESSLER, L.G. (1985). The emerging impact of flow cytometry in predicting recurrence and survival in breast cancer: a review. *J. Natl Cancer Inst.*, **75**, 405.

PETO, R., PIKE, M.C., ARMITAGE, P. *et al.* (1977). Design and analysis of randomized clinical trials requiring prolonged observation of each patient. II. Analysis and observation. *Br. J. Cancer*, **35**, 1.

REDMAN, J.R., PETRONI, G.R., SAIGO, P.E., GELLER, N.L. & HAKES, T. (1986). Prognostic factors in advanced ovarian cancer. *J. Clin. Oncol.*, **4**, 515.

RODENBERG, C.J., CORNELISSE, C.J., HEINTZ, A.M., HERMANS, J. & FLEUREN, G.J. (1987). Tumor ploidy as a major prognostic factor in advanced ovarian cancer. *Cancer*, **59**, 317.

SEIFERT, W.E. & RUDLAND, P.S. (1974). Possible involvement of cyclic GMP in growth control of cultured mouse cells. *Nature*, **248**, 138.

SEROV, S.F., SCULLY, R.E. & SOBIN, L.H. (1973). *International Classification of Tumours*, No. 9. *Histological Typing of Ovarian Tumours*. WHO: Geneva.

TATTERSALL, M.H.N. (1987). Tumour DNA content. In *Ovarian Cancer – the Way Ahead*, Sharp, F. & Soutter, W.P. (eds) p. 79. Royal College of Obstetricians and Gynaecologists: London.

TURNER, G.A., ELLIS, R.D., GUTHRIE, D., LATNER, A.L., ROSS, W.M. & SKILLEN, A.W. (1982). Cyclic GMP in urine to monitor the response of ovarian cancer to therapy. *Br. J. Obstet. Gynaecol.*, **89**, 760.

WEINSTEINSTEIN, Y., CHAMBERS, D.A., BOURNE, H.R. & MEHNAN, K.L. (1974). Cyclic GMP stimulates lymphocyte nucleic acid synthesis. *Nature*, **251**, 352.

WILLIAMS, C.J., WHITEHOUSE, J.M.A., SLEVIN, M. & GOLDING, P. (1985). Cis platinum combination chemotherapy versus chlorambucil for stage III and IV ovarian cancer. Long term results of a randomised study. *Proc. Am. Soc. Clin. Oncol.*, **4**, 116.

ZEILIG, C.E. & GOLDBERG, N.D. (1977). Cell cycle related changes of '3'5 cyclic GMP in Novikoff Hepatoma cells. *Proc. Natl Acad. Sci. USA*, **74**, 1052.